# Family Visiting Restrictions and Postoperative Clinical Outcomes: A Retrospective Analysis

**DOI:** 10.3390/nursrep12030057

**Published:** 2022-08-12

**Authors:** Matteo Danielis, Rosa Iob, Illarj Achil, Alvisa Palese

**Affiliations:** Department of Medical Sciences, University of Udine, Viale Ungheria 20, 33100 Udine, Italy

**Keywords:** visiting policies, surgical patients, clinical outcomes, caregivers, COVID-19

## Abstract

In the last two years, all hospitals have adopted restricted visitation policies due to the coronavirus disease 2019. The objective of this study was to assess the consequences of hospital visitation restrictions on the most common outcome measures on adult patients who underwent surgery. A retrospective study design was conducted according to the STrengthening the Reporting of OBservational studies in Epidemiology statements in 2021. Forty patients exposed to a no-visitors policy and forty unexposed patients (1:1) were enrolled. Patients who were not allowed to receive family visits were more likely to report disorientation/agitation episodes (*n* = 25, 62.5% vs. *n* = 12, 30.0%; *p* < 0.01), spend more sleepless nights (*n* = 10, 25.0% vs. *n* = 1, 2.5%; *p* < 0.01), be restrained (*n* = 8, 20.0% vs. *n* = 1, 2.5%; *p* = 0.02), incur device-removal incidents (*n* = 14, 35.0% vs. *n* = 5, 12.5%; *p* = 0.01) compared to unexposed patients. Conversely, pain episodes were significantly more frequent in the unexposed group (*n* = 7.1, SD = 7.9 vs. *n* = 2.4, SD = 2.8; *p* < 0.01), and there was lower clinical deterioration risk (NEWS of 0–4 average 19.5, SD = 12.2 evaluations vs. 12.3, SD = 8.6; *p* < 0.01) compared to exposed patients. According to the results, family visiting restrictions should be measured against their possible advantages in order to prevent negative outcomes for surgical patients and to improve the quality of care.

## 1. Introduction

The concept of patient-centred care (PCC)—universally recognised as promoting high-quality care [1,2]—has evolved to that of family-centred care (FCC) and of patient and family-centred care (PFCC) gaining relevance in hospital settings globally [3]. Operationally, PFCC as well as FCC include open units, where the care planning, delivery, and evaluation is based on partnerships, allowing family relatives (FRs) to be present at the bedside and to participate in expressing the needs, preferences and values of patients, in detecting the symptoms of patients, and in offering emotional support.

In recent decades, the practice of health professionals has embodied the principles of PFCC and FCC to the point that nurses recognise that the presence of FRs at the bedside has a considerable influence on improving the condition of patients [4]. Hospital policies that allow the presence of FRs have been largely implemented showing their effectiveness among patients with dementia [5], cancer [6], cardiovascular diseases [7], and among frail elderlies [8]. In addition, the physical and psychological conditions of both patients with chronic diseases and nurses improved when family caregivers were involved [9].

In a surgical context, the presence of FRs has become a standard of care [10] with several positive outcomes. In an American retrospective cohort study of 2462 patients undergoing ambulatory surgery and accompanied by caregivers, the presence of a FR contributed to reducing inpatient care duration, anticipating patient discharge, and patients being transferred from outpatient to inpatient care less frequently. In addition, the nurses were more likely to provide psychosocial support and positively contributed to successful patient education and patient satisfaction [11]. In Denmark, a qualitative study of patients in a fast-track colon cancer surgery programme discovered that talking to family members during the postoperative recovery period gave patients the confidence they needed to leave hospital [12]. According to a British qualitative study, patients recovering after fast-track liver resection surgery believed that family support was a key factor in deciding whether to pursue early discharge [13].

Engaging families in surgical wards may help patients to cope with a challenging situation. However, with the intent to ensure the safety of both patients and visitors, family visiting has been suspended in most hospitals during the coronavirus disease 2019 (COVID-19) pandemic [14]. Recent restrictions have disrupted the routine practices of engaging families in the care of their beloveds, with the effects investigated only from the perceptions of patients [15]. To the best of our knowledge, the potential consequences of a no-visitor policy on the outcomes of patients in surgical wards have not yet been documented. Therefore, the main intent of this study was to assess the hypothesis that wards that implemented significant changes—including extensive restrictions on the presence of family—during the pandemic period have compromised the quality of care. Hence, in line with a quantitative approach, the aim of this study was to contribute to the advancement of knowledge regarding the consequences of the restrictions on FRs on postoperative outcomes.

## 2. Materials and Methods

### 2.1. Study Design and Setting

A retrospective cohort study [16] was conducted according to the STrengthening the Reporting of OBservational studies in Epidemiology statements (Table S1) [17]. A postoperative surgical unit (15 beds) of an academic hospital (>1000 beds) ensuring a 1:5 nurse-to-patient ratio was approached. Preliminarily, the homogeneity of care delivered (nurse-to-patient ratio, bed occupancy rate, the competences of nurses) was assessed between two periods, namely, 2019 and 2021.

### 2.2. Data Collection Process

Data were collected from two periods. The exposed patients were selected from patients cared for between 1 March and 30 April 2021, when FRs were prohibited to access the hospital due to COVID-19 pandemic restrictions (P1); the unexposed were patients cared for from 1 March to 30 April 2019, when no restrictions were in place, and visits were allowed 24/7 (P2). In both periods, a stratified random sampling [18] was used: all eligible patients (>18 years) were cared for in the unit for at least 72 h after the urgent or scheduled surgical procedures. Forty exposed patients and forty unexposed patients, who were matched based on age (±9 years), gender, and surgical procedure (1:1) were enrolled. In particular, gastrointestinal (*n* = 40), vascular (*n* = 20), and orthopaedic (*n* = 20) surgery were encompassed: the number of exposed and unexposed patients undergoing each type of surgery was equal.

Based on the literature [5,7,11], outcome measures were established as follows: disorientation/agitation, anxiety, or depressive episodes (number); sleepless nights (number); pain episodes (numerical rating scale > 3); clinical deterioration evaluations (number) measured using the National Early Warning Score (NEWS, episodes of low, medium, and high risk of deterioration) [19]; the use of physical restraints (PRs) (including hours/patient); device-removal incidents (e.g., urinary catheters); pro re nata (PRN) medications; and length of stay (LOS). Outcomes measures, demographics, and some clinical data (e.g., previous history of neurological disorders) were extracted from the medical records of identified patients using a prespecified data collection form.

### 2.3. Data Analysis

Descriptive statistics were performed. Data were analysed using the SPSS version 27, IBM Corp., Armonk, NY, USA by calculating means, standard deviations [SD], frequencies, and percentages. Differences between the groups were compared to t-tests and chi-squared tests (statistical significance set at *p*-Value < 0.05). When the chi-squared tests were performed on observations fewer than five (e.g., depression episodes), the *p*-Values were computed using the bootstrap simulation (Monte Carlo test with 1,000,000 replicates).

### 2.4. Risk-of-Bias Assessment

A 1:1 sampling of exposed and unexposed patients might have introduced a suboptimal statistical efficiency. However, to overcome biases in the sample selection, we matched exposed and unexposed subjects to a number of predefined criteria to ensure that exposed patients were as similar as possible to unexposed patients. To prevent information bias, only data available from medical records were collected. Moreover, data were homogenously collected in medical records forms by nurses caring for patients and trained in data recording; no new record procedures were introduced over the studied periods. To prevent performance bias, data were analysed in a blinded fashion by one author, without knowing whether patients were exposed to family absence or not.

## 3. Results

In total, 40 exposed patients (14 women and 26 men, mean age 72.9 years) and 40 unexposed patients (15 women and 25 men, mean age 73.6 years), who were homogeneous in terms of demographics and clinical characteristics, were included (Table 1). Those patients who were not allowed to receive visits from FRs were more likely to report disorientation/agitation episodes (*n* = 25, 62.5% vs. *n* = 12, 30.0%; *p* < 0.01) and two or more sleepless nights (*n* = 10, 25.0% vs. *n* = 1, 2.5%; *p* < 0.01) compared to unexposed patients. Moreover, they were more likely to be restrained (*n* = 8, 20.0% vs. *n* = 1, 2.5%; *p* = 0.02) and for more total hours (*n* = 7.8 [SD = 23.2] vs. *n* = 0.25 [SD = 1.6]; *p* = 0.05). They were also more likely to incur device-removal incidents compared to unexposed patients (*n* = 14, 35.0% vs. *n* = 5, 12.5%; *p* = 0.01).

Conversely, pain episodes were significantly more frequent in the unexposed group (*n* = 7.1 [SD = 7.9] vs. *n* = 2.4 [SD = 2.8]; *p* < 0.01) as were the administrations of PRN analgesic medications (*n* = 4.9 [SD = 7.2] vs. *n* = 2.8 [SD = 2.6] *p* = 0.10), albeit not significantly compared to exposed patients. Furthermore, the unexposed group reported, on average, a lower clinical deterioration risk than exposed patients (NEWS of 0–4 average 19.5, SD = 12.2 evaluations vs. 12.3, SD = 8.6; *p* < 0.01). No other statistical difference between the exposed and unexposed patients emerged.

## 4. Discussion

To the best of our knowledge, this is the first study investigating the clinical effects associated with restrictions on FRs imposed by the COVID-19 pandemic among postoperative patients.

More than double the number of patients who did not receive visits from FRs manifested disorientation/agitation compared to those who received them, and significantly more of them reported ≥ two sleepless nights. Family caregivers are essential to provide support, psychosocial assistance, and comfort [11] that may prevent agitation and ameliorate sleep patterns at night. Moreover, nearly a quarter of patients who did not receive visits from FRs were physically restrained for more hours; one third of them accidentally removed a device. Bedside relatives have been documented to prevent the application of physical restraints, to promote safety during episodes of agitation, and to prevent the removal of devices [11,15]. Additionally, they might have played a pivotal role in the increase in informal surveillance rates by detecting episodes of agitation and/or the risk of the removal of devices in advance. Due to their in-depth knowledge of the patient, the quality of such surveillance can be considered equally effective to that provided by nurses [20]. Although not measured, the nursing surveillance during the COVID-19 pandemic could have been reduced in its rate to minimise all but essential contact with patients.

From this perspective, an interpretation of why higher averages of pain episodes—and the consequent analgesic medications—among patients with a FR at the bedside occur could be the following: the lowest averages of pain episodes among those left alone might indicate that their pain was not detected and communicated. After all, pain management is considered the primary target in postsurgical care. Similarly, the significant lower averages of evaluations in the low-risk clinical deterioration category (NEWS scores [19]) among patients not receiving visits from FRs compared to unexposed patients may indicate that relatives might play an active role in recognising the clinical deterioration of a patient early. Considering the global nursing shortage—exacerbated by the COVID-19 pandemic—FRs may assume different roles in healthcare delivery [3], such as providing emotional support for a patient, decision-making, and other care roles at the bedside.

Although there was no statistical difference between the two groups, the LOS among the patients not receiving visits from FRs was shorter compared to the counterpart. The consequences of bedside caregivers on LOS among patients with complex needs—including surgical—has not been well documented to date, and the few results available were found to be inconclusive [11]. It can be assumed that the influence of family visiting may have a dual impact on adults recovering from surgery: on the one hand, it may encourage them to undertake rehabilitation activities; on the other, it may create a stressful effect, increasing pressure on both the patient and the staff.

### Limitations

Firstly, a single centre was approached and, therefore, the results cannot be generalised. Additionally, the scope of this study is restricted to just two short periods, thus preventing any analysis of the implications of the pandemic and how infection prevention strategies implemented by the hospital have been revised over time. Secondly, a basic statistical analysis was performed using descriptive methods, without priori estimations of sample size and propensity score evaluation. Moreover, according to the nature of the study design, causality could not be inferred. Furthermore, in addition to the presence/absence of FRs, other different variables between the two time periods may have played a role in the detected differences. For example, the quality of the nursing surveillance might have been affected by the masks also worn by patients. In addition, the differences in the profile of patients (elective vs. urgent cases) may have also affected the results.

## 5. Conclusions

This study provides a preliminary assessment of the consequences of a visitor restriction policy on patients in a surgical ward. The results suggest that the absence of FRs in postoperative settings can lead to negative outcomes, such as disorientation that might turn into sleep disturbances, increased use of physical restraints, and the accidental removal of devices. Conversely, restrictions did not affect pain and clinical deterioration detection and management. Therefore, the restrictions on FRs should be balanced between their potential benefits (e.g., preventing the negative outcomes on patients) and threats (e.g., increasing the spread of the virus).

Future research in several areas is suggested by our study. Firstly, multicentre studies are recommended with a sample size estimated around the expected outcomes (e.g., disorientation/agitation episodes). Secondly, it is advised that nurses continue to gather data on outcomes that are known to be sensitive to FCC/PFCC to enhance our understanding of how COVID-19 restrictions affect the quality of care delivered. An appropriate sample size combined with standardised data collection will establish an increased strength of evidence.

Furthermore, it is imperative to reimplement family visiting when formulating policies in response to potential pandemic exacerbations.

## Figures and Tables

**Table 1:** Demographics, and clinical and outcome variables.

	P1 Exposed (*n* = 40)	P2 Unexposed (*n* = 40)	*p*-Value
***Demographic variables***			
Age (years), mean (SD)	72.9 (16.2)	73.6 (15.2)	0.83
Gender (female), *n* %	14 (35.0)	15 (38.0)	0.81
BMI (kg/m^2^), mean (SD)	25.9 (5.5)	25.9 (6.3)	0.98
***Clinical variables***			
Patients with history of neurological disorder, *n* (%)	8 (20.0)	5 (12.5)	0.36
Patients with history of psychiatric disorder, *n* (%)	2 (5.0)	3 (7.5)	1.00
***Outcome variables***			
Patients with disorientation/agitation episode(s), *n* (%)	25 (62.5)	12 (30.0)	0.00 **
Patients with anxiety episode(s), *n* (%)	4 (10.0)	0 (−)	0.12
Patients with depression episode(s), *n* (%)	5 (12.5)	1 (2.5)	0.20
Patients with sleepless night(s), *n* (%)			
≤1	30 (75.0)	39 (97.5)	0.00 **
≥2	10 (25.0)	1 (2.5)	
Pain episode(s) with NRS > 3/patient, mean (SD)	2.4 (2.8)	7.1 (7.9)	0.00 **
Clinical deterioration,			
Overall NEWS ^†^ score, mean (SD)	2.7 (1.8)	2.7 (1.6)	0.93
NEWS ^†^ 0–4, episode(s)	12.3 (8.6)	19.5 (12.2)	0.00 **
NEWS ^†^ 5–6, episode(s)	1.9 (2.5)	2.4 (3.4)	0.46
NEWS ^†^ ≥7, episode(s)	1.0 (2.9)	1.4 (5.4)	0.72
Patients restrained, *n* (%)	8 (20.0)	1 (2.5)	0.02 *
Hours with physical restraints/patient, mean (SD)	7.8 (23.2) ^‡^	0.25 (1.6)	0.05
Patients with device-removal incident(s), *n* (%)	14 (35.0)	5 (12.5)	0.01 *
Analgesic, PRN medications/patient, mean (SD)	2.8 (2.6)	4.9 (7.2)	0.10
Psychotropic, PRN medications/patient, mean (SD)	0.8 (1.5)	0.7 (1.7)	0.83
LOS (days), mean (SD)	6.2 (3.3)	6.6 (4.4)	0.59

P1: Period 1; P2: Period 2; SD: standard deviation; BMI: body mass index; NEWS: National Early Warning Score; PRN: pro re nata, which means ‘as needed’; LOS: length of stay. *Note*: In P1, patients did not receive visiting FRs according to no-visitor policies; while in P2, patients were allowed to receive FRs 24/7 according to the hospital policies. ^†^ A scoring system based on six common physiological measurements: respiration rate, oxygen saturation, systolic blood pressure, pulse rate, level of consciousness or new confusion, and temperature. A score of 0, 1, 2, or 3 is allocated to each parameter: low risk (aggregate scores from 1 to 4), medium risk (from 5 to 6), and high risk (aggregate scores of 7 or over). ^‡^ Two outlier patients physically restrained from 91 and 106 h, respectively. * *p* < 0.05; ** *p* < 0.01

## Data Availability

Data were obtained from Udine University and are available from the authors on reasonable request.

## Supplementary Materials

The following supporting information can be downloaded at https://www.mdpi.com/article/10.3390/nursrep12030057/s1, Table S1. STROBE Statement—Checklist of items that should be included in reports of cohort studies.

## References

[1] Santana M.J., Manalili K., Jolley R.J., Zelinsky S., Quan H.D., Lu M.S. (2018). How to practice person-centred care: A conceptual framework. Health Expect..

[2] Naik A.D., Catic A. (2021). Achieving patient priorities: An alternative to patient-reported outcome measures (PROMs) for promoting patient-centred care. BMJ Qual. Saf..

[3] Herrin J., Harris K.G., Kenward K., Hines S., Joshi M.S., Frosch D.L. (2021). Patient and family engagement: A survey of US hospital practices. BMJ Qual. Saf..

[4] Shibily F.M., Aljohani N.S., Aljefri Y.M., Almutairi A.S., Almutairi W.Z., Alhallafi M.A., Alsharif F., Almutairi W., Badr H. (2021). The Perceptions of Nurses and Nursing Students Regarding Family Involvement in the Care of Hospitalized Adult Patients. Nurs. Rep..

[5] Barbosa A., Sousa L., Nolan M., Figueiredo D. (2015). Effects of Person-Centered Care Approaches to Dementia Care on Staff: A Systematic Review. Am. J. Alzheimers Dis. Other Demen..

[6] Tremblay D., Roberge D., Touati N., Maunsell E., Berbiche D. (2017). Effects of interdisciplinary teamwork on patient-reported experience of cancer care. BMC Health Serv. Res..

[7] Fors A., Gyllensten H., Swedberg K., Ekman I. (2016). Effectiveness of person-centred care after acute coronary syndrome in relation to educational level: Subgroup analysis of a two-armed randomised controlled trial. Int. J. Cardiol..

[8] Chung P.Y.F., Ellis-Hill C., Coleman P. (2017). Supporting activity engagement by family carers at home: Maintenance of agency and personhood in dementia. Int. J. Qual. Stud. Health Well-Being.

[9] Neumann D., Lamprecht J., Robinski M., Mau W., Girndt M. (2018). Social relationships and their impact on health-related outcomes in peritoneal versus haemodialysis patients: A prospective cohort study. Nephrol. Dial. Transpl..

[10] Gillick M.R. (2013). The Critical Role of Caregivers in Achieving Patient-Centered Care. JAMA.

[11] Griffin S., McGrath L., Chesnut G.T., Benfante N., Assel M., Ostrovsky A., Levine M., Vickers A., Simon B., Laudone V. (2019). Impact of caregiver overnight stay on postoperative outcomes. Int. J. Health Care Qual. Assur..

[12] Burrell B., Trip H. (2011). Recovering at home: Participating in a fast-track colon cancer surgery programme. Nurs. Inq..

[13] Vandrevala T., Senior V., Spring L., Kelliher L., Jones C. (2016). ‘Am I really ready to go home?’: A qualitative study of patients’ experience of early discharge following an enhanced recovery programme for liver resection surgery. Support. Care Cancer.

[14] Hart J.L., Turnbull A.E., Oppenheim I.M., Courtright K.R. (2020). Family-Centered Care During the COVID-19 Era. J. Pain Symptom Manag..

[15] Zeh R.D., Santry H.P., Monsour C., Sumski A.A., Bridges J.F.P., Tsung A., Pawlik T.M., Cloyd J.M. (2020). Impact of visitor restriction rules on the postoperative experience of COVID-19 negative patients undergoing surgery. Surgery.

[16] Song J.W., Chung K.C. (2010). Observational Studies: Cohort and Case-Control Studies. Plast. Reconstr. Surg..

[17] von Elm E., Altman D.G., Egger M., Pocock S.J., Gotzsche P.C., Vandenbroucke J.P., Initiative S. (2014). The Strengthening the Reporting of Observational Studies in Epidemiology (STROBE) Statement: Guidelines for reporting observational studies. Int. J. Surg..

[18] Elfil M., Negida A. (2017). Sampling methods in Clinical Research; an Educational Review. Emergency.

[19] Klepstad P.K., Nordseth T., Sikora N., Klepstad P. (2019). Use of National Early Warning Score for observation for increased risk for clinical deterioration during post-ICU care at a surgical ward. Ther. Clin. Risk Manag..

[20] Puurveen G., Baumbusch J., Gandhi P. (2018). From Family Involvement to Family Inclusion in Nursing Home Settings: A Critical Interpretive Synthesis. J. Fam. Nurs..

