# Supplementary material for: Family Visiting Restrictions and Postoperative Clinical Outcomes: A Retrospective Analysis
Source: Nurs Rep. 2022 Aug 12;12(3):583–8. doi: 10.3390/nursrep12030057 (PMC9397009; doi:10.3390/nursrep12030057)
Supplement: Supplementary file 1 [file nursrep-12-00057-s001.zip › nursrep-1803218-supplementary.pdf]

Table S1. STROBE Statement—Checklist of items that should be included in reports of ***cohort studies***.

	Item n.	Recommendation	Descriptions
**Title and abstract**	1	(*a*) Indicate the study's design with a commonly used term in the title or the abstract	Reported in the title
		(*b*) Provide in the abstract an informative and balanced summary of what was done and what was found	Abstract reported
**Introduction**			
Background/rationale	2	Explain the scientific background and rationale for the investigation being reported	Summarised in the Introduction
Objectives	3	State specific objectives, including any prespecified hypotheses	Reported in the end of the Introduction
**Methods**			
Study design	4	Present key elements of study design early in the paper	The key elements are reported under the Materials and Methods
Setting	5	Describe the setting, locations, and relevant dates, including periods of recruitment, exposure, follow-up, and data collection	Settings (Academic hospital, Post-operative unit), recruitment periods, relevant dates, and data collection, have been all reported in the Materials and Methods
Participants	6	(*a*) Give the eligibility criteria, and the sources and methods of selection of participants. Describe methods of follow-up	Eligibility criteria have been reported in the methods (all postoperative patients (>18 years) cared for in the surgical unit of at least 72 hours after the surgical procedures)
		(*b*) For matched studies, give matching criteria and number of exposed and unexposed	Matching criteria have been reported under the methods (age (± 9 years), gender and surgical procedure group)
Variables	7	Clearly define all outcomes, exposures, predictors, potential confounders, and effect modifiers. Give diagnostic criteria, if applicable	Outcome measures were detailed in the methods (disorientation/agitation, anxiety, depression episodes; sleepless nights; pain episodes, clinical deterioration; PRs use, device-removed incidents; PRN medications and in the LOS)
Data sources/ measurement	8*	For each variable of interest, give sources of data and details of methods of assessment (measurement). Describe comparability of assessment methods if there is more than one group	Outcomes measures, demographic and some clinical data were extracted from the medical records of identified patients
Bias	9	Describe any efforts to address potential sources of bias	These efforts have been described in the risk-of-bias assessment paragraph
Study size	10	Explain how the study size was arrived at	A sample size calculation was not

			performed
Quantitative variables	11	Explain how quantitative variables were handled in the analyses. If applicable, describe which groupings were chosen and why	Reported in the methods
Statistical methods	12	(*a*) Describe all statistical methods, including those used to control for confounding	Reported in the methods (data were analysed with the SPSS version 27, IBM Corp., Armonk, NY, USA by calculating means, standard deviations [SD], frequencies and percentages)
		(*b*) Describe any methods used to examine subgroups and interactions	Not performed
		(*c*) Explain how missing data were addressed	No missing data have been detected in the medical records among that considered in this study: data were collected on a routine basis according to the minimum data set established at the hospital level
		(*d*) If applicable, explain how loss to follow-up was addressed	Not applicable
		(*e*) Describe any sensitivity analyses	Not applicable
**Results**			
Participants	13*	(a) Report numbers of individuals at each stage of study—eg numbers potentially eligible, examined for eligibility, confirmed eligible, included in the study, completing follow-up, and analysed	Summarised in the results. All consecutive eligible exposed and unexposed were considered and, when satisfying the inclusion criteria, included
		(b) Give reasons for non-participation at each stage	Not required given that all consecutive eligible exposed and unexposed were assessed for eligibility and then included
		(c) Consider use of a flow diagram	Not required given that all consecutive eligible exposed and unexposed were assessed for eligibility and then included
Descriptive data	14*	(a) Give characteristics of study participants (eg demographic, clinical, social) and information on exposures and potential confounders	Characteristics of participants are summarised in the results and in the Table 1
		(b) Indicate number of participants with missing data for each variable of interest	No missing data have been detected in the medical records among that considered in this study: data were collected on a routine basis as a minimum data set established at the hospital level
		(c) Summarise follow-up time (eg, average and total amount)	Not applicable
Outcome data	15*	Report numbers of outcome events or	Reported in the results

		summary measures over time	
Main results	16	(*a*) Give unadjusted estimates and, if applicable, confounder-adjusted estimates and their precision (eg, 95% confidence interval). Make clear which confounders were adjusted for and why they were included	A basic statistical analysis has been performed by using descriptive statistics (mean, SD, frequencies and percentages); then, differences between the groups were compared with t-tests and chi-squared tests
		(*b*) Report category boundaries when continuous variables were categorized	Not performed
		(*c*) If relevant, consider translating estimates of relative risk into absolute risk for a meaningful time period	Not performed
Other analyses	17	Report other analyses done—eg analyses of subgroups and interactions, and sensitivity analyses	Not performed
**Discussion**			
Key results	18	Summarise key results with reference to study objectives	Reported in the discussion
Limitations	19	Discuss limitations of the study, taking into account sources of potential bias or imprecision. Discuss both direction and magnitude of any potential bias	Reported in the discussion (limits)
Interpretation	20	Give a cautious overall interpretation of results considering objectives, limitations, multiplicity of analyses, results from similar studies, and other relevant evidence	Reported in the discussion
Generalisability	21	Discuss the generalisability (external validity) of the study results	Reported in the discussion (limits)
**Other information**			
Funding	22	Give the source of funding and the role of the funders for the present study and, if applicable, for the original study on which the present article is based	Not applicable

PR, Physical Restraint; PRN, Pro Re Nata; LOS, Length of Stay; SD, Standard Deviation.

*Give information separately for exposed and unexposed groups.

Note: An Explanation and Elaboration article discusses each checklist item and gives methodological background and published examples of transparent reporting. The STROBE checklist is best used in conjunction with this article (freely available on the Web sites of PLoS Medicine at http://www.plosmedicine.org/, Annals of Internal Medicine at http://www.annals.org/, and Epidemiology at http://www.epidem.com/). Information on the STROBE Initiative is available at http://www.strobe-statement.org.
